# The Bradley–Terry Regression Trunk approach for Modeling Preference Data with Small Trees

**DOI:** 10.1007/s11336-022-09882-6

**Published:** 2022-09-03

**Authors:** Alessio Baldassarre, Elise Dusseldorp, Antonio D’Ambrosio, Mark de Rooij, Claudio Conversano

**Affiliations:** 1 University of Cagliari, Cagliari, Italy; 2 Leiden University, Leiden, Netherlands; 3 University of Naples Federico II, Naples, Italy

**Keywords:** paired comparisons, preference rankings, regression tree, STIMA, GLM

## Abstract

This paper introduces the Bradley–Terry regression trunk model, a novel probabilistic approach for the analysis of preference data expressed through paired comparison rankings. In some cases, it may be reasonable to assume that the preferences expressed by individuals depend on their characteristics. Within the framework of tree-based partitioning, we specify a tree-based model estimating the joint effects of subject-specific covariates over and above their main effects. We, therefore, combine a tree-based model and the log-linear Bradley-Terry model using the outcome of the comparisons as response variable. The proposed model provides a solution to discover interaction effects when no a-priori hypotheses are available. It produces a small tree, called trunk, that represents a fair compromise between a simple interpretation of the interaction effects and an easy to read partition of judges based on their characteristics and the preferences they have expressed. We present an application on a real dataset following two different approaches, and a simulation study to test the model’s performance. Simulations showed that the quality of the model performance increases when the number of rankings and objects increases. In addition, the performance is considerably amplified when the judges’ characteristics have a high impact on their choices.

The analysis of preference data is ubiquitous in many scientific fields. Preferences are analyzed in several ways, depending on how these are collected from a set of individuals, or judges. People can express their preferences with respect to a set of items (or stimuli, or objects) by assigning a numerical value to each of them according to an ordinal scale or can place in order the objects by forming a list, called ordering, in which the preferences are stated by looking at the order in which each object appears in the list (Marden, 1996).

Sometimes objects are presented in pairs to judges, producing the so-called paired comparison rankings: This could be the natural experimental procedure when the objects to be ranked are really similar and the introduction of other objects may be confusing (David, 1969). Given a ranking of $n_o$ objects, it is possible to determine the set of $n_o\times (n_o-1)/2$ pairwise preferences, but this set does not always correspond to a ranking because of the phenomenon of non-transitivity of the preferences. This phenomenon could be avoided by ensuring that ‘individuals comparisons are independent or nearly’ (David, 1969, p. 11).

In analyzing rank data, the goal is often to find one ranking that best represents all the preferences stated by each individual. This goal, when dealing with rank vectors, is known as the consensus ranking problem, the Kemeny problem, or the rank aggregation problem (Amodio et al., 2016). When dealing with paired comparison rankings, the goal is to determine the probability that object *i* is preferred to object *j* for all the possible pairs of them: The final outcome is thus a probabilistic assessment of the central ranking (Kendall & Babington Smith, 1940; Bradley & Terry, 1952; Mallows, 1957).

Preference rankings can be analyzed with both supervised and unsupervised methods. Among these, there are methods based on the goodness-of-fit adaptation aimed at describing the structure of rank data (Coombs, 1950; Carroll, 1972; Meulman et al., 2004; Busing et al., 2005; D’Ambrosio et al., 2021) and probabilistic methods (Marden, 1996; Heiser & D’Ambrosio, 2013) that assume a homogeneous or heterogeneous distribution of judges preferences. When homogeneity is assumed, probabilistic methods are based on the so-called Thurstonian models (Thurstone, 1927). Heterogeneity of preferences implies that different groups of subjects with specific characteristics may show different preference rankings (Strobl et al., 2011) and is accounted for introducing subject-specific covariates from which mixtures of known sub-populations can be estimated, in most cases, with generalized linear models (Chapman & Staelin, 1982; Dittrich et al., 2000; Böckenholt, 2001; Francis et al., 2002; Skrondal & Rabe-Hesketh, 2003; Gormley & Murphy, 2008) or recursive partitioning methods (i.e., tree-based) (Strobl et al., 2011; Lee & Yu, 2010; D’Ambrosio & Heiser, 2016, Plaia & Sciandra, 2019).

Dittrich et al. (2000) proposed a parametric model for the analysis of rank ordered preference by means of Bradley–Terry (BT)-type models with categorical subject-specific covariates. They transform the (complete) rankings data into paired comparisons and apply a log-linear model for a corresponding contingency table. The search for the interaction effects between covariates is based on a forward selection and backward elimination procedure. Although this approach is suited for hypothesis-based modeling, it requires an effective selection of the covariates and a distinct choice of the functional form in which these covariates are added to the model (Strobl et al., 2011). Thus, it requires the arbitrary introduction of higher-order interactions when no a priori hypotheses are known.

Strobl et al. (2011) proposed a tree-based classifier, where the paired comparisons are treated as response variables in Bradley-Terry models. They found a way to discover interactions when no a priori hypothesis is known, suggesting a model-based recursive partitioning where splits are selected with a semi-parametric approach by looking for instability of the basic Bradley–Terry model object parameters. The final result provides the preference scales in each group of the partition that derives from the order of object-related parameters, but it does not offer information about how the subject-specific covariates affect the judges’ preferences. Thus, this semi-parametric model returns parametric coefficients neither for the main effects nor for the interaction effects.

Recently, Wiedermann et al. (2021) extended the Strobl’s model by combining the log-linear Bradley–Terry (LLBT) model with the model-based recursive partition (MOB) for detecting treatment effect heterogeneity. They proposed a semi-parametric model that distinguishes between focal independent variables and covariates for recursive partition. A score-based procedure, the M-fluctuation test (Zeileis & Hornik, 2007, 2008), is used to assess the stability of model parameters, and the pruning procedure is conducted using the AIC.

In this paper, we propose a completely parametric approach that tries to overcome the drawbacks of the models introduced in Dittrich et al. (2000) and Strobl et al. (2011). It fits a generalized linear model with a Poisson distribution by combining its main effects with a parsimonious number of interaction effects. Our approach is framed within the simultaneous threshold interaction modeling algorithm (STIMA) proposed by Dusseldorp et al. (2010) and Conversano & Dusseldorp (2017) that, in the case of a numerical response, is based on the regression trunk approach (Dusseldorp & Meulman, 2004). Dealing with paired comparisons, it combines the extended log-linear Bradley–Terry model including subject-specific covariates with the regression trunk. Thus, the proposed model is named *Bradley-Terry regression trunk (BTRT)*. BTRT produces an estimated generalized linear model with a log link and a Poisson distribution presenting a main effects part and an interaction effects part, the latter being composed of a restricted number of higher-order interactions between covariates that are automatically detected by the STIMA algorithm. The interaction effect part can be graphically represented in a decision tree structure, called trunk, because it is usually characterized by few terminal nodes. Hence, BTRT allows observing the preference scale in each node of the trunk and to evaluate how the probability of preferring specific objects changes for different groups of individuals. The final result is a small tree that represents a compromise between the interpretability of interaction effects and the ability to summarize the available information about the judges’ preferences.

The main feature of BTRT is that it does not require a selection of the covariates to be added to the model nor a specification of their functional form. Moreover, its output provides a specific estimated parameter for the variables composing the main effects part of the model as well as for the possible interactions between subject-specific covariates. The differences with respect to the Wiedermann et al. model are due to the different split search procedures based on the MOB model. As pointed out by the authors, the testing procedure for the split search can be very challenging. They use the M-fluctuation test to search for the best splitting covariate, while our method is based on the easy-to-compute decrease in deviance introduced in the regression trunk approach within the STIMA algorithm. Both methods can deal with continuous or categorical subject-specific covariates, even if the current implementation of BTRT does not handle nominal covariates. Furthermore, as in the Wiedermann et al. model, also in the STIMA algorithm it is possible to distinguish between focal predictors and partitioning covariates, choosing the treatment variable as the first split variable.

The rest of the paper is organized as follows. In Sect. 1, we give an overview of the basic Bradley–Terry model and its extension with subject-specific covariates. Next, the STIMA algorithm and the regression trunk methodology are recalled in Sect. 2 before introducing BTRT and explaining how it can efficiently be used for the task of partitioning individuals based on their preferences. A simulation study has been carried out to investigate, in particular, the choice of a suitable pruning rule: results are reported in Sect. 3. In Sect. 4, we present an application of BTRT on a real dataset. Conclusions and future research directions are reported in Sect. 5.

## The (Extended) Bradley–Terry Model

The Bradley–Terry model [BT, Bradley & Terry, 1952] derives a latent preference scale from paired comparison data when no natural measuring scale is available. It has been applied in psychology and several other disciplines (Dittrich et al., 2006; Choisel & Wickelmaier, 2007; Rodríguez Montequín et al., 2020).

Let $\pi _{(ij)i}$ denote the probability that the object *i* is preferred in the comparison with *j*. The probability that *j* is preferred is $\pi _{(ij)j} = 1-\pi _{(ij)i}$. The basic Bradley–Terry model can be defined as (Agresti, 2002, p. 436-439)

1 $$\begin{aligned} \pi _{(ij)i} = \frac{\pi _i}{\pi _i + \pi _j}, \end{aligned}$$

where $\pi _i$ and $\pi _j$ are nonnegative parameters (also called worth parameters) describing the location of objects on the preference scale. Eq. (1) can be expressed as a logistic model for paired preference data. With a set of $n_o$ objects to be judged, by following Sinclair (1982) for which

2 $$\begin{aligned} \pi _{(ij)i} = \frac{\pi _i}{\pi _i + \pi _j} = \frac{\sqrt{\pi _i / \pi _j }}{ \sqrt{\pi _i / \pi _j } + \sqrt{\pi _j / \pi _i } }, \end{aligned}$$

the BT model can be defined as a quasi-symmetry model for paired comparisons with object parameters $\lambda _i^O$ such that

3 $$\begin{aligned} logit(\pi _{(ij)i}) = \log \left( \frac{\pi _{(ij)i}}{\pi _{(ij)j}}\right) = \lambda _i^O - \lambda _j^O, \end{aligned}$$

where $\lambda _i^O$ and $\lambda _j^O$ are object parameters related to $\pi$’s in Eq. (2) by $\lambda _i^O = \frac{1}{2}\ln (\pi _i)$. The superscript *O* refers to object-specific parameters. Thus, ${\hat{\pi }}_{(ij)i} = \frac{\exp {({\hat{\lambda }}_i^O - {\hat{\lambda }}_j^O)}}{1+\exp {({\hat{\lambda }}_i^O - {\hat{\lambda }}_j^O)}}$, where $\pi _{(ij)i} = \frac{1}{2}$ when $\lambda _i^O = \lambda _j^O$. The model estimates $$\left( {\begin{array}{c}n_o\\ 2\end{array}}\right) $$ probabilities, which is the number of paired comparisons with $n_o$ objects. Note that the logit model in Eq. (3) is equivalent to the model in Eq. (1). Identifiability of the two models requires a restriction on the parameters related to the last object $n_o$, such as $\lambda _{n_o}^O = 0$ or $\sum _i^{n_o} \pi _i$ = 1.

The BT model can also be fitted as a log-linear model (Fienberg & Larntz, 1976; Sinclair, 1982; Dittrich et al., 1998). Sinclair (1982) assumed that, in comparing object *i* with object *j*, the random variables $y_{(ij)i}$ and $y_{(ij)j}$ follow a Poisson distribution and represent the number of times a specific comparison occurs. Let $n_{ij}$ be the number of comparisons made between object *i* and *j*, and $m(y_{(ij)i})$ be the expected number of comparisons in which *i* is preferred to *j*. Then, combining the re-specification proposed by Sinclair and the notation for log-linear models for contingency tables, it follows that, $m(y_{(ij)i}) = n_{ij}\pi _{(ij)i}$ has a log-linear representation and, conditional on the fixed marginal total, its distribution is multinomial

4 $$\begin{aligned} \begin{aligned} \log (m(y_{(ij)i}))&= \mu _{ij} + \lambda _i^O - \lambda _j^O \\ \log (m(y_{(ij)j}))&= \mu _{ij} - \lambda _i^O + \lambda _j^O. \end{aligned} \end{aligned}$$

The nuisance parameters $\mu$ in Eq. (4) may be interpreted as interaction parameters representing the objects involved in the respective comparison, therefore fixing the corresponding $n_{ij}$ marginal distributions (Dittrich et al., 2004; Dittrich & Hatzinger, 2009). In total, $$2\left( {\begin{array}{c}n_o\\ 2\end{array}}\right) $$ expected counts are estimated. This approach allows synthesizing the information about all preferences in a unique design matrix. The columns of the design matrix represent the responses $y_{(ij)}$, the parameter $\mu$ expressed as a factor indicating the $n \times (n-1) / 2$ comparisons, and the object parameters $\lambda _i^O$. An example of design matrix for three objects is given in Table 11 in the Appendix.

When $y_{(ij)}$ assumes values $+1$ and $-1$ instead of 1 and 0, respectively, the linear predictor $\eta$ of the basic log-linear BT model is (Hatzinger & Dittrich, 2012)

5 $$\begin{aligned} \eta _{y_{(ij)i}} = \log (m(y_{(ij)i})) = \mu _{ij} + y_{(ij)i}(\lambda _i^O - \lambda _j^O). \end{aligned}$$

Equation (5) can be extended by introducing multiple subject-specific covariates. For continuous subject-specific covariates it is necessary to build up a separate contingency table for each judge, and each different value of the covariate. An example in which two judges, with different ages, express their preferences regarding three objects is shown in Table 12 in the Appendix. For a categorical covariate *S*, let $m(y_{(ij)i,l})$ be the expected number of preferences for *i* compared with *j*, among individuals classified in covariate category *l*, with $l = 1 \dots L$, where *L* represents the total number of levels of the covariate. The BT model is then specified as

6 $$\begin{aligned} \begin{aligned} \log (m\left( y_{(ij)i,l}\right) )&= \mu _{ij,l} + \lambda _i^O - \lambda _j^O + \lambda _l^S + \lambda _{i,l}^{OS} - \lambda _{j,l}^{OS} \\ \log (m\left( y_{(ij)j,l}\right) )&= \mu _{ij,l} - \lambda _i^O + \lambda _j^O + \lambda _l^S - \lambda _{i,l}^{OS} + \lambda _{j,l}^{OS}, \end{aligned} \end{aligned}$$

where $\lambda _l^S$ is the main effect of the subject-specific covariate *S* measured on its *l*-th level; $\lambda _{i,l}^{OS}$ and $\lambda _{j,l}^{OS}$ are the subject-object interaction parameters describing the effect of *S* observed on category *l* and concerning the preference for object *i* and *j*, respectively. If *S* has no effect on the preferences of the judges, then $\lambda _{i,l}^{OS} = 0$ and the model collapses into the previously described basic BT model: There is just one log-odds for the comparison of two specific objects (Hatzinger & Dittrich, 2012). The parameters of interest $\lambda _{i,l}^{OS}$ and $\lambda _{j,l}^{OS}$ in Eq. (6) can still be interpreted as log-odds and log-odds ratio

7 $$\begin{aligned} \log \left( \frac{\pi _{(ij)i,l}}{\pi _{(ij)j,l}}\right) = 2(\lambda _i^O + \lambda _{il}^{OS}) - 2(\lambda _j^O + \lambda _{jl}^{OS}). \end{aligned}$$

Hence, the LLBT equation for the *h*-th judge and objects *i* and *j* is

8 $$\begin{aligned} \log (m\left( y_{(ij)i,h}\right) ) = \mu _{ij,h} + y_{(ij)i,h}(\lambda _{i,h}^O-\lambda _{j,h}^O). \end{aligned}$$

The parameter $\lambda _{i,h}^O$ can be expressed through a linear relation

9 $$\begin{aligned} \lambda _{i,h}^O = \lambda _i^O + \sum _{p = 1}^P \beta _{ip}x_{p,h}, \end{aligned}$$

where $\lambda _i^O$ (intercept) indicates the location of object *i* in the overall consensus ranking, $x_{p,h}$ is the value of the $x_p$-th continuous covariate $(p = 1,\ldots ,P)$ observed for judge *h* and $\beta$ measures the effect of $x_p$ on object *i*.

The deviance of the model in Eq. (7) indicates how well the model fits the data. It corresponds to the deviance of a fitted Poisson regression

10 $$\begin{aligned} D=2 \sum _{h=1}^H y_{ij,h} \times \log \left( \frac{y_{ij,h}}{m(y_{ij,h})}\right) , \end{aligned}$$

where $y_{ij,h}$ represents the observed values of each comparison *ij* for each judge *h*, and $m(y_{ij,h})=\hat{y}_{ij,h}$ are the predicted values based on the estimated model parameters. If the model fits well, the $y_{ij,h}$ will be close to their predicted values $m(y_{ij,h})$.

## The Bradley–Terry Regression Trunk (BTRT) for Preference Data

The BT model is hereby applied to preference data by specifying a regression model for paired comparisons. This specification is aimed at estimating, in an automatic and data-driven fashion, both the main effects and, if present, the interaction effects part of the model. For this purpose, we resort to the STIMA framework extended with the use of GLM in Conversano & Dusseldorp (2017) and combine the extended BT model including subject-specific covariates with the regression trunk methodology (Dusseldorp & Meulman, 2004). The latter allows the user to evaluate in a unique model the importance of both main and interaction effects by first growing a regression trunk and then by pruning it back to avoid overfitting. The interaction effects are hereby intended as a particular kind of non-additivity (Berrington de González & Cox, 2007; Cohen et al., 2013).

STIMA integrates generalized linear models—GLM (McCullagh & Nelder, 1989) and classification and regression trees (CART) (Breiman et al., 1984), and is used when the analyst has no exact a priori hypotheses about the nature of the interaction effects (e.g., in Conversano et al., 2019). Notationally, the GLM estimated by STIMA assumes that a response variable *y* observed on *n* subjects has an exponential family density $\rho _y(y;\theta ;\phi )$ with a natural parameter $\theta$ and a scale parameter $\phi$. The response *y* depends on a set of *P* categorical and/or continuous covariates $x_p$ ($p=1,\ldots ,P)$ and its mean $\mu = E(y|x_1,\ldots ,x_P)$ is linked to the $x_p$s via a link function $g(\cdot )$:

11 $$\begin{aligned} g(\mu ) = \eta = \beta _0 + \sum _{p=1}^P \beta _p x_{p,h} + \sum _{t = 1}^{T-1} \beta _{P+t} I\{(x_{1,h},\ldots ,x_{P,h}) \in t \} \end{aligned}$$

Equation (11) refers to a standard GLM presenting a linear predictor $\eta$ such that $\mu = g^{-1}(\eta )$ ($\mu$ is an invertible and smooth function of $\eta$). The first *P* parameters concern the main effects part of the model estimated in the root node of the trunk via standard GLM, while the other $T-1$ parameters define the interaction effects part of the model obtained by partitioning recursively in a binary way the *n* cases in order to add additional interaction terms defined by the coefficients $\beta _{P+t }$ and the indicator variables $I\{(x_{1,h},\ldots ,x_{P,h}) \in t \}$. Being obtained by a sequential binary splitting of the original data, the interaction effects correspond to threshold interactions since the values/labels of the splitting predictors leading to a specific terminal node can be considered as thresholds that partition the predictor space in order to correctly identify a GLM with interaction effects that maximizes goodness of fit by controlling for overfitting.

The Bradley–Terry regression trunk (BTRT) model combines the extended log-linear BT model including subject-specific covariates (Eqs. 8 and 9) with the STIMA-based trunk model (Eq. 11). In BTRT, the estimated consensus expressed for object *i* by the judge *h* is

12 $$\begin{aligned} {{\hat{\lambda }}}_{i,h} = {{\hat{\lambda }}}_i + \sum _{p=1}^P \hat{\beta }_{i,p} x_{p,h} + \sum _{t = 1}^{T-1} {{\hat{\beta }}}_{i,P+t} I\{(x_{1,h},\ldots ,x_{P,h}) \in t \}, \end{aligned}$$

in which the subscript *O* is left out from the notation of the ${\hat{\lambda }}$ parameters for readability reasons. Again, the term $\sum _{p=1}^P {{\hat{\beta }}}_{i,p} x_{p,h}$ is the main effects part assessing the effects of covariates on the consensus for object *i*. The interaction effects part is estimated by $\sum _{t = 1}^{T-1} {{\hat{\beta }}}_{i,P+t} I\{(x_{1,h},\ldots ,x_{P,h}) \in t \}$ and is derived from the terminal nodes of a regression trunk that searches for possible threshold interactions between the *P* covariates assuming that they have a joint effect on the consensus expressed for object *i* besides their individual (main) effect. Thus, the regression trunk has *T* terminal nodes and for each terminal node *t* an additional parameter $\beta _{i,P+t}$ is estimated. It expresses the effect of the threshold interaction between the covariates $x_1,\ldots ,x_P$ whose split points lead to *t*. The estimated intercept term ${{\hat{\lambda }}}_i$ measures the average consensus about object *i* in the root node of the trunk while the estimated intercept for the terminal node *t* is ${\hat{\lambda }}_i + {\hat{\beta }}_{i, P+t}$. The model in Eq. (12) is still a log-linear model aimed at modeling the pairwise comparisons of objects *i* and *j* (Eq. 8) through a different specification of the linear components describing the consensus expressed for the objects (see Eq. 9 for object *i*).

Although the estimation procedure of BTRT is framed within the STIMA algorithm, some steps are different. Once a set of paired comparisons is given, a preliminary data processing step is required to obtain the design matrix of the BT model. In our framework, ties are not included, but the model can be extended by incorporating undecidedness parameters. The final design matrix is composed of $n=n_o \times (n_o-1) \times H$ rows, where *H* indicates the number of judges. The total number of rows is equal to the product between the number of comparing objects, that is 2, the number of paired comparisons ($n_o \times (n_o-1)/2)$, and the number of judges, resulting in $2 \times (n_o \times (n_o-1)/2) \times H$.

### Growing the Bradley–Terry Regression Trunk

In each step of STIMA, a generalized linear model with a Poisson link is fitted to the data. To discover the main effects, it is only necessary to fit the model in the root node. The first estimated model consists of *P* $\beta$ coefficients that describe the probability distribution of preferring a particular object to another one, given a set $(x_1,...,x_P)$ of judges’ characteristics. The search for the best split of the trunk at each iteration is made by taking into account all the available terminal nodes at that step. For a particular terminal node and based on paired comparisons, for each covariate $x_p$, with $(p=1,\ldots P)$, we consider each unique value of $x_{p}$ as a candidate split point. Specifically, a Bradley-Terry model is estimated for each of the possible pairs of candidate values $ij \in [1,n_o]; i \ne j$, by discretizing $x_p$ and creating the associated dichotomous variable $z_{ijp}$.

Next, the split point associated with $z^*_{ijp}$ maximizing the decrease in deviance is computed with respect to the goodness-of-fit test based on the deviance of a Poisson regression model introduced in Eq. (10). Thus, it is considered as the ‘best’ split point and the node is split according to the specific value of the discretized variable $x_p$. The splitting criterion of BTRT is based on maximizing the decrease in deviance when moving from a parent node to the two possible daughter nodes defined by splitting on $z_{ijp}$. This split search procedure is repeated by searching for each splitting node *t* the best split point so that, once found, the new dichotomous variable $z^*_{ijp,t}$ is added to the model and an additional interaction effect is included. When the split is found, all regression coefficients in the model are re-estimated.

Preliminarily, the user is required to choose between two main approaches that could be followed in BTRT:

a *One Split Only (OSO)*, where the splitting covariates already used in the previous splits are not considered as candidate splitting variables for the current split;
b *Multiple Splitting (MS)*, where the whole set of covariates is considered to split the current node despite some of them have been previously selected to split other nodes.

The OSO approach returns a tree in which it is possible to analyze the interaction effects between all the covariates. Since, in this case, a covariate cannot split two subsequent nodes of the tree, the risk of possible ‘spurious interactions’ is avoided. In this case, the final tree might not necessarily return the best model as that producing the best goodness of fit (i.e., maximum reduction in deviance). Besides, following the MS approach it is possible to achieve the maximum reduction in deviance, but there is a risk of obtaining a tree that utilizes the same covariate (with different values) to split several, even subsequent, nodes. In this case, it can happen that only the main effects part is retained and thus it is not possible to analyze interactions. We compare the two criteria in the real data application (see Sect. 4).

At each split step, the estimated regression parameters $\hat{\beta }_{i,P+t}$ measure the probability of preferring a specific object *i*, given the interaction between different characteristics of a particular group of judges. While some similar methods, such as M5 (Quinlan, 1992) and Treed regression (Alexander & Grimshaw, 1996), estimate several linear models, one in each node of the tree, the regression trunk model estimates a single linear model only.

**Fig. 1:**
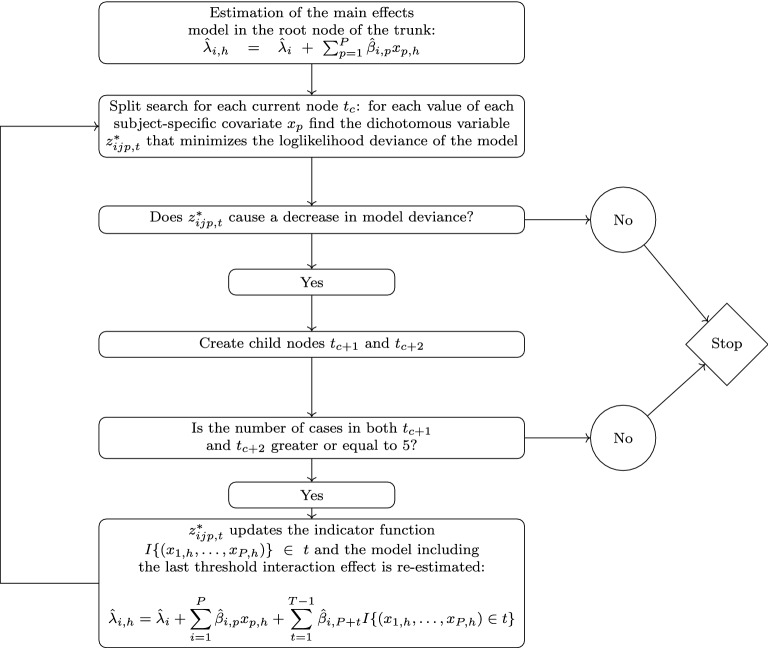
Flowchart of the STIMA algorithm implementing the BTRT model for preference data.

Consistent with standard criteria applied in decision tree modeling, the stopping criterion of BTRT is based on the a-priori definition of the minimum number of observations for a node to be split. The default implementation is based on the requirement that the size of the new nodes should be at least equal to five, even if the minimum bucket size can be modified based on the depth of the tree requested by the user. Figure 1 shows a flowchart in which the tree growing procedure is schematically explained.

The final BTRT model estimates a number of parameters equal to the number of intercepts, plus the number of main effects parameters, plus the number of interactions. The total number of parameters is computed as follows:

13 $$\begin{aligned} (n_o - 1) + [P \times (n_o - 1)] + [(T-1) \times (n_o - 1)]. \end{aligned}$$

### Pruning the Bradley–Terry Regression Trunk

When the final estimated trunk model presents a large number of higher-order interactions, it may be challenging to interpret the results and the overfitting problem might occur. Anyway, growing the maximum expanded trunk is necessary since a small trunk may not be able to capture the real interactive structure of the data if the splitting process ends too early. For this reason, BTRT considers a pruning procedure operated after the trunk growing. In particular, a *V*-fold cross-validation of the BTRT model deviance is computed for each step split of the trunk. The user has to provide the number of subsets *V* in which the entire dataset is divided. The minimum sample size requirements for the choice of *V* depends on the number of judges, the number of objects to be compared and the number of subject-specific covariates, which all determine the dimension of the design matrix. As there is not a formal rule to follow, we recommend to decrease the number of folds of the CV procedure and possibly repeat the CV procedure several times (i.e., m times *V*-fold cross-validation), if the number of judges and/or the number of comparing objects is limited. To obtain the cross-validated deviance, all the preferences expressed by a particular judge *h* in the design matrix are randomly assigned to a specific subset and, for *V* times, the BTRT trunk model estimated in a specific node is trained on $V-1$ subsets while the left-out subset is treated as a test set. At the end of the process, a predicted value ${\hat{y}_{ij,h}}$ is obtained for each observation in the data matrix. Following this approach, the case-wise cross-validation deviance $D^{cv}$ is

14 $$\begin{aligned} D^{cv} =\frac{1}{n} \left[ 2 \sum _{i'=1}^n y_{i'j;h}\times \log \left( \frac{y_{i'j;h}}{\hat{y}_{i'j;h}}\right) \right] , (i',j) \in n_o, (i'\ne j), h \in H \end{aligned}$$

where *n* is equal to the total number of rows of the design matrix and $i'$ is its generic row. Note that the number of rows *n* is greater than the total number of judges *H*. The standard error of $D^{cv}$ is

15 $$\begin{aligned} SE^{cv} = \sqrt{ \frac{1}{n} \sum _{i'=1}^n \left[ y_{i'j;h} \times \log \left( \frac{y_{i'j;h}}{\hat{y}_{i'j;h}}\right) - D^{cv} \right] ^2}. \end{aligned}$$

Usually, $$D^{cv}$$ decreases after the first splits of the trunk and starts to increase next. BTRT uses the same $$c \cdot SE$$ pruning rule used in STIMA (Dusseldorp et al., 2010). Let $$t^* \in [1,T]$$ be the size of the regression trunk with the lowest $$D^{cv}$$, say $$D^{cv}_{t^*}$$. The best size of the BTRT trunk $$t^{**}$$ corresponds to the minimum value of *t* such that $$D^{cv}_{t^{**}} \le D^{cv}_{t^*} + c \cdot SE^{cv}_{t^*}$$.

## Simulation Study: The Choice of the Pruning Parameter

Pruning the BTRT model with the *c* $\cdot$ SE rule requires the choice of the most suitable value for the parameter *c*. The optimal value may depend on characteristics of the data, such as sample size (Dusseldorp et al., 2010). In this section, a simulation study is carried out to assess the value of the optimal *c* to be used to select the final BTRT model.

For the regression trunk approach used to detect threshold interactions in the linear model, Dusseldorp et al. (2010) reported that most of the times a value of $c = 0$ results in a regression trunk with too many interaction terms while a value of $c = 1$ gives a small-sized regression trunk with too few interaction terms.

As for BTRT, we compare the performance of seven pruning rules obtained by specifying seven different values of *c* ranging from 0 to 1, namely 0.00, 0.10. 0.30, 0.50, 0.70, 0.90 and 1.00.

Three different scenarios are considered for the data generating process (DGP):

16 $$\begin{aligned}{} & {} \lambda _{i,h} = \lambda _i + \beta _{i,1}x_{1,h}; \end{aligned}$$

17 $$\begin{aligned}{} & {} \lambda _{i,h} = \lambda _i + \sum _{p=1}^4 \beta _{i,p}x_{p,h}; \end{aligned}$$

18 $$\begin{aligned}{} & {} \lambda _{i,h} = \lambda _i + \sum _{p=1}^4 \beta _{i,p}x_{p,h} + \beta _{i,5}I(x_{1,h}> 0.00 \cap x_{2,h} > 0.50). \end{aligned}$$

In the first scenario (Eq. 16), only one subject-specific covariate ($x_1$) affects the preferences expressed by the generic judge *h* on each object *i*. In the second one (Eq. 17), four subject-specific covariates are assumed to influence the judges’ preferences. These two models present linear main effects only so that the performance metric of the pruning rules is the proportion of times a BTRT model with at least one interaction term is selected (type I error). In the third scenario (Eq. 18), a model including both linear main effects and threshold interaction effects is considered as a threshold interaction term between $x_1$ and $x_2$ is added to the main effects part of the model. In this case, the performance metric of the pruning rule is the type II error, obtained by computing the proportion of times the selected regression trunk model does not include $x_1$ and $x_2$ exactly as the first and only two interacting variables. In all cases, all the covariates $x_p$ are standard normally distributed.

### Design Factors and Procedure

Three design factors are considered in the simulation study:

- The number of judges *H*: 100, 200, 300;
- The number of objects $n_o$: 4, 5. The consensus rankings were set as (A B C D) and (A B C D E), respectively, by using decreasing values of $\lambda _i$, namely (0.9, 0.4, 0.3, 0.0) in the first case, and (0.8, 0.4, 0.2, 0.1, 0.0) in the second one;
- The effect size of each covariate $x_p$ on the preferences expressed by the judge *h* on each object *i*. Values of the parameters $\beta _i$ are reported in Table 1 for each set of objects, the two possible effect sizes and the three different scenarios.

**Table 1:** Simulated values of $\beta _i$ for the estimation of the pruning parameter *c*.

Effect-size	Low				High					
object	A	B	C	D	A	B	C	D		
N. objects = 4										
1st scenario (Eq. 16)										
$\beta _1$	0.30	0.20	0.10	0.00		0.90	0.80	0.70	0.00	
2nd scenario (Eq. 17): add $\beta _2$, $\beta _3$ and $\beta _4$										
$\beta _2$	0.20	0.30	0.10	0.00		0.80	0.70	0.90	0.00	
$\beta _3$	0.10	0.20	0.30	0.00		0.70	0.90	0.80	0.00	
$\beta _4$	0.30	0.10	0.20	0.00		0.90	0.70	0.80	0.00	
3rd scenario (Eq. 18): add $\beta _5$										
$\beta _5$	0.25	0.15	0.35	0.00		0.55	0.65	0.45	0.0	
N. objects = 5										
1st scenario (Eq. 16)										
$\beta _1$	0.40	0.30	0.20	0.10	0.00	0.90	0.80	0.70	0.60	0.00
2nd scenario (Eq. 17): add $\beta _2$, $\beta _3$ and $\beta _4$										
$\beta _2$	0.30	0.20	0.10	0.40	0.00	0.80	0.90	0.60	0.70	0.00
$\beta _3$	0.20	0.10	0.30	0.40	0.00	0.70	0.60	0.80	0.90	0.00
$\beta _4$	0.10	0.20	0.40	0.30	0.00	0.90	0.70	0.60	0.80	0.00
3rd scenario (Eq. 18): add $\beta _5$										
$\beta _5$	0.25	0.15	0.35	0.45	0.00	0.55	0.65	0.45	0.60	0.00

We only considered the case of 4 and 5 objects as design factors because working on paired comparisons means extending the number of judges’ evaluations to 6 and 10, respectively. It seems more realistic that only few objects are presented to judges when working on paired comparisons. Furthermore, as the number of objects increases, the size of the design matrix increases, as does the computational cost of searching for the split. However, the computational cost does not increase in the same way when the number of judges increases. For this reason, the BTRT model is not computationally expensive when the number of judges is high, whereas the computational time increases as long as the number of objects increases. The combination of the three design factors ($n_o \times H \times$ *effect size*) results in 12 different BTRT specifications. For each of them, we generated 100 random samples, so that 1,200 datasets were generated for each true scenario, given in Eqs. (16), (17), and (18). In each run, a BTRT with a maximum of five terminal nodes (*T* = 5) is estimated.

Once the design factors are set, following Eq. 1 the values of ${\hat{\lambda }}_{i,h}$ are estimated in order to obtain the probability that a judge *h* prefers the object *i* to *j*. The latter are computed for each possible comparison as follows

19 $$\begin{aligned} \pi _{(ij)i,h} = \frac{\exp {[2({\hat{\lambda }}_{i,h}-{\hat{\lambda }}_{j,h})]}}{1+\exp {[2({\hat{\lambda }}_{i,h}-{\hat{\lambda }}_{j,h}})]}; \end{aligned}$$

The design matrix of the log-linear Bradley Terry model requires the values of *y* in the first column. The response *y* is coded as a 0–1 variable depending on whether or not an individual preference occurs for each comparison *ij*. Thus, we consider $y_{ij,h}$ as the realization of a Bernoulli distribution that assumes the value 1 with probability $\pi _{(ij)i,h}$. The main problem for this kind of coding is that it is possible to obtain combinations of 0-1 values for the same judge that do not verify the transitivity property between the preferences. The number of all possible combinations of two values for each judge is equal to $2^{\frac{n_o(n_o-1)}{2}}$, where the exponent is the number of paired comparisons obtainable from $n_o$ objects. However, when ties are not allowed, the number of permutations of $n_o$ objects is equal to $n_o!$, which is much smaller than the number of all the possible combinations of two values. When $n_o$ is higher than 3, it is very likely to obtain combinations that do not find a counterpart in the universe of allowed rankings. For instance, when the number of objects is equal to four, there could be 64 different combinations of 0–1 values, of which only 24 are allowed. Thus, there could be 40 not allowed combinations. To avoid this problem, we replaced these not allowed combinations with the closest permutation in the universe of $n_o!$ rankings.

### Results

Results of the simulation study are summarized in Tables 2, 3 and 4. For the first two scenarios, the pruning rules are evaluated with respect to the type I error (Tables 2, 3) while for the third scenario the focus is on the type II error (Table 4). To facilitate the interpretation of the results, the tables for type II error show the power of the pruning rules (i.e., 1 - type II error), rather than the type II errors. Results are reported for the 9 different values of the *c* parameter (0, 0.1, 0.3, 0.5, 0.7, 0.9, 1), as well as for the number of objects (4 or 5), the number of judges (100, 200 or 300) and the effect sizes (Low or High). As conventionally done, a threshold value of 0.05 is used for type I error (probability of incorrectly identifying an interaction effect). Hence, higher values are shown in boldface because type I error is considered too large. For power we used the value 0.8 as threshold so that a value less than 0.8 is considered unsatisfactory and thus reported in boldface.

Table 2 reports the results for the first scenario where only the main effects of the single covariate $x_1$ are considered. When the number of objects is equal to 4 and the effect of $x_1$ is low, the pruning rules with $c \ge 0.3$ result in acceptable type I errors despite the sample size. However, when the effect size increases, the case with $H = 100$ requires higher values of *c* (i.e., $c \ge 0.7$) for the pruning parameter. When the number of objects is equal to 5 the inverse situation is observed: For small effect sizes higher values of *c* (i.e., $c \ge 0.7$) are required, while for a high effect sizes lower values of *c* (i.e., $c \ge 0.5$) can be used.

**Table 2:** Results first scenario: type I error. Error higher than 0.05 in boldface.

N. objects	$n_o = 4$						$n_o = 5$					
Effect size	Low			High			Low			High		
N. judges	100	200	300	100	200	300	100	200	300	100	200	300
c = 0.0	**0.76**	**0.82**	**0.82**	**0.95**	**1.00**	**1.00**	**0.80**	**0.90**	**0.98**	**0.75**	**0.84**	**0.82**
c = 0.1	**0.16**	**0.18**	0.04	**0.62**	**0.51**	**0.58**	**0.60**	**0.58**	**0.60**	**0.30**	**0.38**	**0.26**
c = 0.3	0.01	0.00	0.00	**0.26**	**0.12**	**0.08**	**0.32**	**0.18**	**0.28**	**0.08**	**0.08**	0.00
c = 0.5	0.00	0.00	0.00	**0.08**	0.05	0.02	**0.12**	0.04	**0.10**	0.00	0.02	0.00
c = 0.7	0.00	0.00	0.00	0.03	0.00	0.00	0.04	0.02	0.00	0.00	0.00	0.00
c = 0.9	0.00	0.00	0.00	0.00	0.00	0.00	0.02	0.02	0.00	0.00	0.00	0.00
c = 1.0	0.00	0.00	0.00	0.00	0.00	0.00	0.02	0.02	0.00	0.00	0.00	0.00

Table 3 displays the type I errors when all the covariates $x_1,...,x_4$ influence judges’ preferences individually (second scenario). In this case, for $n_o=4$ the values of $c \ge 0.5$ provide acceptable error rates despite the effect size; for $n_o=5$ and high effect size it would be better to choose a pruning parameter $c \ge 0.7$.

**Table 3:** Results second scenario: type I error. Error higher than 0.05 in boldface.

N. objects	$n_o = 4$						$n_o = 5$					
Effect size	Low			High			Low			High		
N. judges	100	200	300	100	200	300	100	200	300	100	200	300
c = 0.0	**0.88**	**0.86**	**0.98**	**0.95**	**0.94**	**0.98**	**0.97**	**1.00**	**0.98**	**0.91**	**0.96**	**1.00**
c = 0.1	**0.58**	**0.56**	**0.66**	**0.67**	**0.66**	**0.74**	**0.74**	**0.86**	**0.86**	**0.62**	**0.70**	**0.80**
c = 0.3	**0.14**	**0.06**	**0.10**	**0.11**	0.04	**0.10**	**0.09**	**0.14**	**0.12**	**0.16**	**0.28**	**0.18**
c = 0.5	0.04	0.02	0.00	0.01	0.00	0.00	0.01	0.02	0.04	**0.06**	**0.06**	0.02
c = 0.7	0.02	0.00	0.00	0.00	0.00	0.00	0.00	0.00	0.00	0.01	0.00	0.00
c = 0.9	0.00	0.00	0.00	0.00	0.00	0.00	0.00	0.00	0.00	0.01	0.00	0.00
c = 1.0	0.00	0.00	0.00	0.00	0.00	0.00	0.00	0.00	0.00	0.00	0.00	0.00

The third scenario reflects the case in which all the covariates $x_1,...,x_4$ have an influence on the expressed preferences, and the first two covariates interact with each other, as shown in Eq. 18. The power (1 - type II error) is displayed in Table 4 for each possible value of *c*. It emerges that for $n_o=4$ a value of $c\ge 0.3$ is considered as satisfactory despite the effect size (except in case there are 100 judges and low effect size), while for the $n_o=5$ case with high effect size it is preferable to increase the value of *c* up to 0.9.

**Table 4:** Results third scenario: test’s power (1-type II error). Power lower than 0.80 in boldface.

N. objects	$n_o = 4$						$n_o = 5$					
Effect size	Low			High			Low			High		
N. judges	100	200	300	100	200	300	100	200	300	100	200	300
c = 0.0	**0.00**	**0.00**	**0.00**	**0.03**	**0.02**	**0.01**	**0.02**	**0.00**	**0.01**	**0.00**	**0.00**	**0.02**
c = 0.1	**0.45**	**0.52**	**0.28**	**0.30**	**0.20**	0.80	**0.22**	**0.06**	**0.01**	**0.28**	**0.12**	**0.02**
c = 0.3	**0.79**	0.94	0.84	0.84	0.84	0.99	0.82	**0.52**	**0.46**	**0.74**	**0.28**	**0.14**
c = 0.5	0.99	0.99	0.99	0.92	0.94	0.98	0.96	0.96	0.88	0.98	**0.44**	**0.24**
c = 0.7	1.00	1.00	1.00	0.96	0.98	1.00	1.00	1.00	1.00	0.98	0.80	**0.56**
c = 0.9	1.00	1.00	1.00	1.00	1.00	1.00	1.00	1.00	1.00	1.00	1.00	0.90
c = 1.0	1.00	1.00	1.00	1.00	0.98	1.00	1.00	1.00	1.00	1.00	1.00	0.96

Recall that low values of the parameter *c* may return a large tree. In the first two scenarios, the true model does not include interaction between variables, so low *c* parameter values return a too high type I error. In the third scenario, the true model refers to a tree of minimum size with a single interaction. For this reason, as the effect size of the covariates and the population size increase, higher values of parameter *c* are required to obtain a high power. It follows that the ability of the BTRT model to find the right interactions between covariates increases when the number of judges and objects increases. In addition, if the judges’ characteristics have a high impact on the choices, then the quality of performance of the BTRT model improves considerably.

Summarizing, results of the simulation study show that a value of the pruning parameter *c* between 0.5 and 1 is a good choice in almost all situations. These results are consistent with those reported in Dusseldorp et al. (2010) for the linear regression model and in Conversano & Dusseldorp (2017) for the logistic regression model and should be considered as guidelines by researchers interested in applying BTRT to real data.

## Application on a Real Dataset

In this section, we show a practical application of the regression trunk for preference rankings on a real dataset following two different approaches. The STIMA algorithm based on the BTRT model has been implemented in the *R* environment (R Core Team, 2021) by using the packages *prefmod* (Hatzinger & Dittrich, 2012) and *BradleyTerry2* (Turner & Firth, 2012).

The analyzed data have been collected through a survey carried out at University of Cagliari (Italy). In particular, 100 students ($H = 100$) enrolled in the first year of Master Degree in Business Economics were asked to order five characteristics of an ideal professor ($n_o = 5$) based on what they considered the most relevant: clarity of exposition ($o_1$), availability of teaching material before the lectures ($o_2$), scheduling of midterm tests ($o_3$), availability of slides and teaching material accompanying the selected books ($o_4$), helpfulness of the professor ($o_5$). These characteristics were ranked with values from 1 to 5, where 1 was assigned to the characteristic considered as the most important, and 5 to the least important one. Students were not allowed to indicate ties. Moreover, for each student, seven subject-specific covariates have been collected: year of study ($x_1$), total number of ECTS obtained ($x_2$), grade point average ($x_3$), course attendance in percentage ($x_4$), daily study hours ($x_5$), gender ($x_6$), and age ($x_7$). Table 5 reports the key statistics for each numerical subject-specific covariate. The distribution of the covariate ‘gender’ is: male = $44\%$, female = $56\%$.

**Table 5:** Descriptive statistics of the subject-specific covariates in application.

	Vars	n	Mean	sd	Median	Trimmed	Mad	Min	Max	Range	Skew	Kurtosis	se
Year of study	$x_1$	100	1.18	0.39	1.00	1.10	0.00	1.00	2.00	1.00	1.64	0.70	0.04
ECTS	$x_2$	100	37.69	40.22	27.00	28.89	5.93	0.00	163.00	163.00	1.90	2.23	4.02
Grade point average	$x_3$	100	23.02	6.93	24.80	24.49	3.26	0.00	30.00	30.00	−2.36	5.17	0.69
Course attendance	$x_4$	100	87.37	13.34	90.00	89.53	13.34	40.00	100.00	60.00	−1.22	0.93	1.33
Daily study hours	$x_5$	100	3.73	1.62	4.00	3.64	1.48	0.25	8.00	7.75	0.48	0.05	0.16
Age	$x_7$	100	21.00	3.25	20.00	20.27	1.48	19.00	41.00	22.00	3.16	13.59	0.33

To apply the Bradley–Terry model, the rankings were converted into ten paired comparisons. Dealing with a small number of judges and several covariates, each judge will likely have at least one characteristic that differs from the other judges. In this framework, for each pair of comparing objects the response variable *y* is binary and takes values of 0 and 1. Therefore, 20 observations are obtained for each judge so that the total number of rows *n* is equal to 2000.

Once the design matrix is obtained, a Poisson regression model is estimated in the root node. Next, the split search as described in Sect. 2.1 is performed. In the following, we compare the results obtained for the two splitting options currently implemented for BTRT: the OSO approach and the MS approach.

### One-Split-Only (OSO) Approach

Based on the OSO approach, the full tree can have a maximum number of splits equal to the number of subject-specific covariates *P*. Thus, the maximum depth regression trunk has 7 splits. In this application, the unpruned trunk is composed of 6 splits and 7 terminal nodes as no more splits agreed with the minimum bucket condition (i.e., number of judges greater or equal to five). Table A1 and Fig. A1 in Appendix report the information about the full (unpruned) trunk.

Table 6 reports the node splitting information and the deviance *D* of the final model estimated in each node (see Eq. 10). Notice that the deviance of the main effects model is reported in the first row of Table 6 while the deviance of the model including a simple dichotomous variable inducing the first split of the trunk (*bestsplit1*) is reported in the second row. The threshold interactions are specified starting from the third row of the table, i.e., from *bestsplit2* onwards.

**Table 6:** Pruned regression trunk: OSO approach. The table shows the node in which the split is found, the splitting covariate, and its split point together with the deviance associated with each estimated model.

	Node n.	Splitting covariate	Split Point	Model Deviance
		Main effects (no splits)		1115
bestsplit1	1	$x_3$ (grade point average)	27.50	1096
bestsplit2	2	$x_7$ (age)	25.00	1080
bestsplit3	4	$x_2$ (n. of ECTS)	39.00	1064

The maximum-depth regression trunk is pruned applying the $c \cdot SE$ rule described in Sect. 2.2 based on both the case-wise 10-fold cross-validation deviance ($D^{cv}$) introduced in Eq. 14 and its standard error ($SE^{cv}$, Eq. 15). Table 7 shows the results of the cross-validation estimates.

**Table 7:** 10-fold cross-validation results with OSO approach: $$D = $$ model deviance (Eq. 10); $$D^{cv} = $$ casewise cross-validation deviance (Eq. 14); $$SE^{cv} = $$ standard error of $$D^{cv}$$ (Eq. 15).

	*D*	$D^{cv}$	$SE^{cv}$
mod0	1115	0.5963	0.0006
mod1	1096	0.5914	0.0006
mod2	1080	0.5869	0.0007
mod3	1064	0.5864	0.0007
mod4	1058	0.5881	0.0008
mod5	1048	0.5890	0.0008
mod6	1033	0.5895	0.0008

**Fig. 2:**
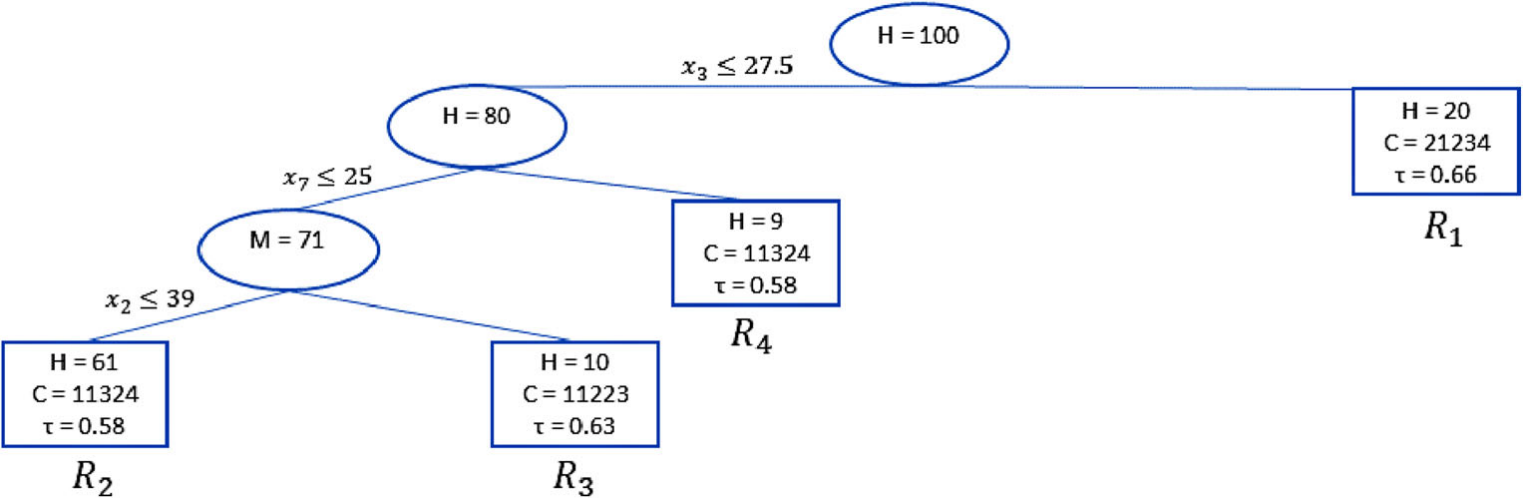
Pruned regression trunk: OSO approach.

Note that $D^{cv}$ is much smaller than the model deviance *D*, because we used two different specifications for these two (see Eqs. 10 and 14): *D* decreases between one model and another, while $D^{cv}$ is decreasing up to the model 3 having four terminal nodes. The pruning rule with the *c* parameter is not necessary in this case, because the cross-validation deviance starts to increase from the fourth model (*mod*4). Thus, the pruned trunk corresponds to the model in Table 6. The final trunk including three splits and $T = 4$ terminal nodes is shown in Fig. 2 .

Figure 2 shows the pruned regression trunk. It reports the number of judges *H* belonging to each terminal node *T*. The consensus ranking *C* is computed by using the differential evolution algorithm for median ranking detection (D’Ambrosio et al., 2017) and the $\tau _x$ rank correlation coefficient (Emond & Mason, 2002) within the group, which measures the strength of the consensus ranking. Both measures are computed using the R package *ConsRank* (D’Ambrosio et al., 2019). The consensus ranking reports the positions of the objects ordered from $o_1$ to $o_5$. Ties are allowed only for the consensus ranking within the groups so that two tied objects have the same associated value. For example, in the terminal node $R_1$ in Fig. 2 the quantity C = 21234 indicates that item $o_1$ is ranked at the second place in a tie with item $o_3$, item $o_2$ is ranked at the first place, and items $o_4$ and $o_5$ are ranked at the third and fourth position, respectively.

### Multiple Splitting (MS) approach

The MS approach allows covariates already used in previous splits to be considered for subsequent splits. To compare the MS approach with the OSO one, a regression trunk with the same number of terminal nodes as the OSO trunk is grown for the MS case (*T* = 7). Results of the full trunk are reported in Table A2 and Figure A2 in the Appendix. Those concerning the pruned trunk are reported in Table 8.

**Table 8:** Pruned regression trunk: MS approach. The table shows the node in which the split is found, the splitting covariate, and its split point together with the deviance associated with each estimated model.

	Node	Covariate	Point	Deviance
		Main effects (no splits)		1115
bestsplit1	1	$x_3$ (grade point average)	27.50	1096
bestsplit2	2	$x_7$ (age)	25.00	1080
bestsplit3	4	$x_2$ (n. of ECTS)	39.00	1064
bestsplit4	8	$x_3$ (grade point average)	21.00	1050

The pruning procedure is based on the 10-fold cross-validation estimation of the deviance and its standard error. Table 9 shows the trunk pruning results obtained from the MS approach.

**Table 9:** 10-fold cross-validation results with MS approach: $$D = $$ model deviance (Eq. 10); $$D^{cv} = $$ casewise cross-validation deviance (Eq. 14); $$SE^{cv} = $$ standard error of $$D^{cv}$$ (Eq. 15).

	*D*	$D^{cv}$	$SE^{cv}$
mod0	1115	0.5963	0.0006
mod1	1096	0.5914	0.0006
mod2	1080	0.5869	0.0007
mod3	1064	0.5864	0.0007
mod4	1050	0.5813	0.0007
mod5	1038	0.5810	0.0008
mod6	1026	0.5812	0.0008
mod7	1018	0.5811	0.0008

**Fig. 3:**
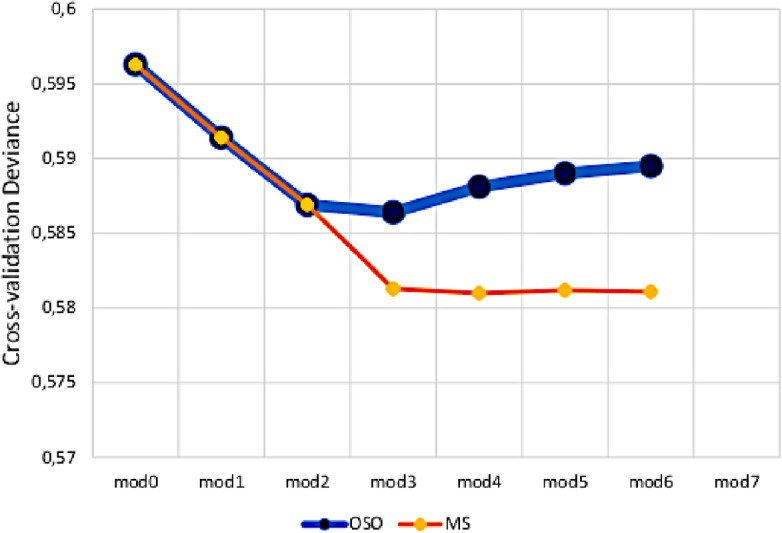
Comparison between OSO and MS approaches.

The MS approach, for each split, generates a reduction in deviance greater than that obtained with the OSO approach. The cross-validation deviance is decreasing up to model 5. Figure 3 compares the two approaches in terms of cross-validation deviance obtained from one split to another. It clearly displays that the MS approach returns a regression trunk capable of better explaining the preferences expressed by the judges.

**Fig. 4:**
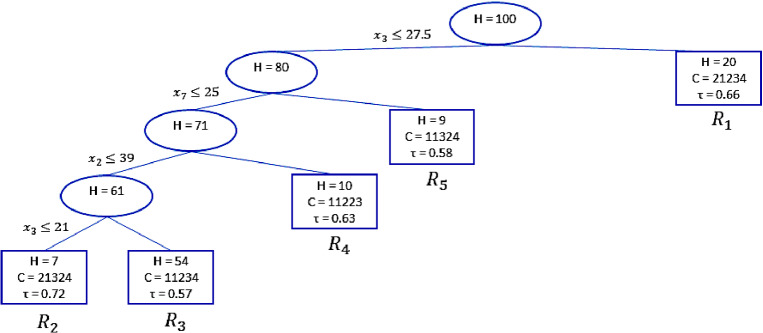
Pruned regression trunk: MS approach.

We consider the results of the simulation study (Sect. 3) with $n_o = 5$ and $H=100$. A possible pruning parameter is $c = 0.5$ so that the final trunk corresponds to model 4 (*mod4*) in Table 9 and is represented in Fig. 4.

Note that in the pruned tree the professor’s quality of exposition ($o_1$) is always preferred to all the other objects, except by the judges in region 1 and 2. As expected, the two approaches provide different results: The OSO approach detects the interaction between all the variables under study, but does not return the best regression trunk in terms of goodness of fit. The MS approach returns a trunk that fits the data better but the final BTRT model may be more challenging to interpret.

The model deriving from the MS regression trunk returns the coefficients shown in Table 10.

**Table 10:** MS regression trunk final output: the table shows the estimated coefficients associated to the objects $o_1$, $o_2$, $o_3$, and $o_4$. The last object $o_5$ is set as reference level, so that the estimated parameters associated to ${\hat{\lambda }}_{o_5,h}$ (the professor helpfulness) are automatically set to zero. The standard errors are shown in parenthesis. There are two standard errors for each parameter: The first is the standard error coming for the Poisson regression, the second one is corrected for the detected overdispersion, which is equal to 1.25.

	${\hat{\lambda }}_{o_1,h}$	${\hat{\lambda }}_{o_2,h}$	${\hat{\lambda }}_{o_3,h}$	${\hat{\lambda }}_{o_4,h}$
${\hat{\lambda }}_i$	3.36 (1.98; 2.22)	4.96 (1.68; 1.88)	3.46 (1.59; 1.78)	−2.41 (1.72; 1.93)
${\hat{\beta }}_{i,x1}$	−0.90 (0.42; 0.48)	−0.43 (0.40; 0.45)	−0.03 (0.40; 0.45)	−0.56 (0.42; 0.47)
${\hat{\beta }}_{i,x2}$	0.02 (0.005; 0.006)	0.009 (0.004; 0.005)	0.003 (0.004; 0.005)	0.009 (0.004; 0.005)
${\hat{\beta }}_{i,x3}$	−0.16 (0.04; 0.05)	−0.14 (0.04; 0.04)	−0.09 (0.03; 0.04)	−0.01 (0.04; 0.04)
${\hat{\beta }}_{i,x4}$	−0.008 (0.006; 0.008)	−0.01 (0.006; 0.007)	-0.01 (0.006; 0.007)	-0.007 (0.006; 0.007)
${\hat{\beta }}_{i,x5}$	−0.04 (0.06; 0.07)	−0.07 (0.05; 0.06)	−0.12 (0.05; 0.06)	−0.06 (0.05; 0.06)
${\hat{\beta }}_{i,x6}$	0.31 (0.18; 0.20)	0.29 (0.15; 0.17)	0.29 (0.15; 0.17)	0.36 (0.15; 0.17)
${\hat{\beta }}_{i,x7}$	0.17 (0.06; 0.07)	0.03 (0.04; 0.05)	0.03 (0.04; 0.05)	0.15 (0.04; 0.05)
${\hat{\beta }}_{i,R2}$	−2.30 (0.62; 0.69)	−1.96 (0.56; 0.63)	−1.47 (0.55; 0.62)	−0.47 (0.59; 0.67)
${\hat{\beta }}_{i,R3}$	−0.90 (0.30; 0.34)	−0.64 (0.25; 0.28)	−0.42 (0.24; 0.27)	0.32 (0.26; 0.29)
${\hat{\beta }}_{i,R4}$	−2.86 (0.58; 0.65)	−1.37 (0.47; 0.53)	−0.73 (0.45; 0.51)	−0.32 (0.46; 0.52)
${\hat{\beta }}_{i,R5}$	−3.56 (0.67; 0.75)	−1.47 (0.53; 0.69)	−1.14 (0.52; 0.58)	−1.32 (0.54; 0.60)

The regions $R_2,\ldots ,R_5$ obtained from the regression trunk represented in Fig. 4 are defined as follows:

$$\begin{aligned}&R_2 = I(\text {grade point average} \le 21, \text {age} \le 25, \text {n. of ECTS} \le 39),\\&R_3 = I(21 < \text {grade point average} \le 27.5, \text {age} \le 25),\\&R_4 = I(\text {grade point average} \le 27.5, \text {age} \le 25, \text {n. of ECTS}> 39 ),\\&R_5 = I(\text {grade point average} \le 27.5, \text {age} > 25), \end{aligned}$$

The region $R_1$ plays the role of reference category. It is defined by the indicator function $I(\text {grade point average} > 27.5)$. From the main effects side, looking at the values in Table 10 the final model shows that the covariates $x_3$ (grade point average) and $x_4$ (course attendance in percentage) have a negative effect on the preferences expressed. In particular, looking at the ${\hat{\beta }}_{i,x_3}$ coefficients, it can be seen that as the grade point average increases, the tendency to prefer the professor’s clarity ($o_1$) to his helpfulness ($o_5$) is lower. On the contrary, it seems that when the number of ECTS increases, the tendency to prefer the professor’s clarity to the professor’s helpfulness is higher. These two results might suggest that for students looking for a high average grade it is very important to interact with professors even outside of the class schedule. On the other hand students who have a high number of ECTS may not be interested in a high average grade, but only in obtaining a degree quickly, hence they recognize as more important the clarity of presentation of topics covered in the class.

As for the interaction effects, looking at Table 10, the last region $R_4$ has a negative coefficients whatever the considered object. In each case, when the students’ grade point average is lower than 27.5 and the age is higher than 25, there is a strong tendency to prefer the professor helpfulness to all other attributes.

## Conclusions

This paper introduces a new Bradley–Terry Regression Trunk (BTRT) model to analyze preference data. BTRT is based on a probabilistic approach in which the judges’ heterogeneity is taken into account with the introduction of subject-specific covariates.

The combination of the log-linear Bradley–Terry model with the regression trunk methodology allows generating, through Poisson regressions, an easy to read partition of judges based on their characteristics and the preferences they have expressed.

The main effects on the object choice of the judges’ characteristics and their interactions are simultaneously estimated. BTRT accounts for the drawback of the classic tree-based models when no a priori hypotheses on the interaction effects are available. At the same time, it allows detecting threshold interactions in an automatic and data-driven mode. The final result is a small and easily interpretable tree structure, called regression trunk, that only considers the interactions that bring relevant improvements to the main effects model fit.

Simulations showed that the ability of the BTRT model to find the right interactions increases when both the sample size and the number of objects to be judged increase, particularly if the covariates have a high impact on the choices. The results suggest that in most of the cases a value of the pruning parameter *c* between 0.7 and 0.9 is a good choice. These values are consistent with those reported in Dusseldorp et al. (2010) for the linear regression model and in Conversano & Dusseldorp (2017) for the logistic regression model.

The two different approaches that have been introduced for the BTRT model have both been used in a real dataset application. It emerges that the One-Split-Only approach aims to verify the interaction effect between all the covariates taken into consideration and the final result is easier to interpret. On the other hand, the Multiple Splitting approach yields a tree more capable of capturing the most relevant interactions between the variables selected by the model.

The BTRT model appears well-suited to analyze the probability distribution of preferring a particular object for a specific group of individuals with a specific set of characteristics. For this reason, it can be used for both descriptive and predictive purposes as it allows the user to estimate the impact of each subject-specific covariate on the judges’ choices, the overall consensus ranking, and the effect size of the interactions between covariates.

Future research is addressed to consider cases when categorical subject-specific covariates with more than two categories are used as possible split candidates as well as to investigate further model performance and stability with respect to (big) datasets presenting a high number of objects, rankings, and covariates. This would allow to better evaluate the two approaches illustrated in Sect. 4. Last but not least, an R package including the function developed to estimate the BTRT parameters and complementary functions to summarize the output and to predict new cases is currently under development.

At the same time, research efforts will be aimed at extending the model to cases where missing values (i.e., partial orderings) are allowed. As the number of objects increases, paired comparisons become more difficult to treat. For this reason, future research may also be oriented to the extension of the BTRT model for the analysis of ordinal data treated as rankings, using not only information relating to the judges, but also the characteristics of the objects themselves (i.e., object-specific covariates).

## Figures and Tables

**Fig. 5:**
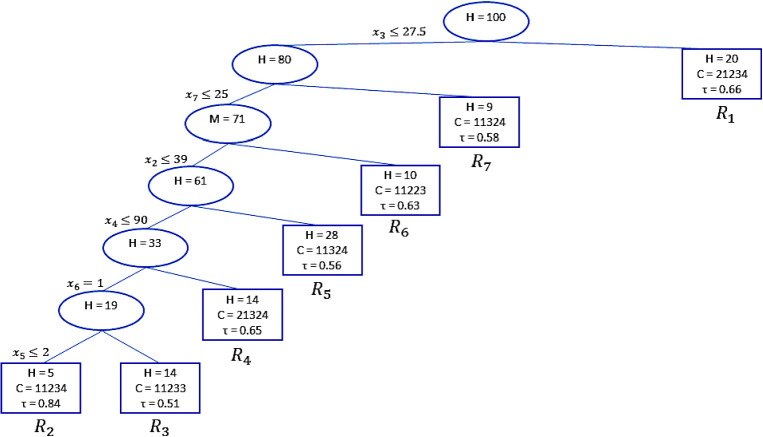
Full regression trunk: OSO approach.

**Fig. 6:**
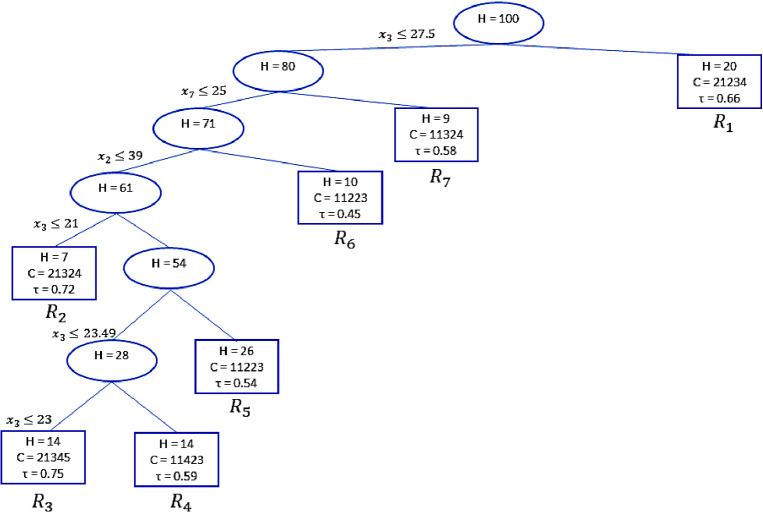
Full regression trunk: MS approach.

## Appendix

**Table 11:** Design matrix with one judge and three objects: The first column indicates the number of times a specific preference is expressed for each pair of objects *ij*. The second column, the parameter $\mu$, serves as an index for the $n \times (n-1) / 2$ comparisons. Finally, preferences are expressed in the last three columns. For example, the first line shows that object *B* is preferred to *A* since $y_{ij} = 1$, $\lambda _B^O = 1$, and $\lambda _A^O = -1$.

Response	$\mu$	$\lambda _A^O$	$\lambda _B^O$	$\lambda _C^O$
$y_{AB} = 1$	1	−1	1	0
$y_{AB} = 0$	1	1	−1	0
$y_{AC} = 1$	2	−1	0	1
$y_{AC} = 0$	2	1	0	−1
$y_{BC} = 1$	3	0	1	−1
$y_{BC} = 0$	3	0	−1	1

**Table 12:** Design matrix with two judges, three objects, and one continuous subject-specific covariate: The first column indicates the number of times a specific preference is expressed for each pair of objects *ij*. The second column serves as an index for the $n \times (n-1) / 2$ comparisons. Preferences are expressed in the next three columns, and finally the age covariate is showed in the last column. In this example, the two judges express opposite preference, BCA and ACB, respectively.

Response	$\mu$	$\lambda _A^O$	$\lambda _B^O$	$\lambda _C^O$	*age*
$y_{AB} = 1$	1	−1	1	0	23
$y_{AB} = 0$	1	1	−1	0	23
$y_{AC} = 1$	2	−1	0	1	23
$y_{AC} = 0$	2	1	0	−1	23
$y_{BC} = 1$	3	0	1	−1	23
$y_{BC} = 0$	3	0	−1	1	23
$y_{AB} = 0$	1	−1	1	0	24
$y_{AB} = 1$	1	1	−1	0	24
$y_{AC} = 0$	2	−1	0	1	24
$y_{AC} = 1$	2	1	0	−1	24
$y_{BC} = 0$	3	0	1	−1	24
$y_{BC} = 1$	3	0	−1	1	24

**Table 13:** Full regression trunk: OSO approach. The table shows the node in which the split is found, the splitting covariate, and its split point together with the deviance associated with each estimated model.

	Node n.	Splitting covariate	Split Point	Model Deviance
		Main effects (no splits)		1115
bestsplit1	1	$x_3$ (grade point average)	27.50	1096
bestsplit2	2	$x_7$ (age)	25.00	1080
bestsplit3	4	$x_2$ (n. of ECTS)	39.00	1064
bestsplit4	8	$x_4$ ($\%$ course attendance)	90	1058
bestsplit5	16	$x_6$ (gender)	1.00	1048
bestsplit6	32	$x_5$ (daily study hours)	2.00	1033

**Table 14:** Full regression trunk: MS approach. The table shows the node in which the split is found, the splitting covariate, and its split point together with the deviance associated with each estimated model.

	Node	Covariate	Point	Deviance
		Main effects (no splits)		1115
bestsplit1	1	$x_3$ (grade point average)	27.50	1096
bestsplit2	2	$x_7$ (age)	25.00	1080
bestsplit3	4	$x_2$ (n. of ECTS)	39.00	1064
bestsplit4	8	$x_3$ (grade point average)	21.00	1050
bestsplit5	17	$x_3$ (grade point average)	23.49	1038
bestsplit6	34	$x_3$ (grade point average)	23.00	1026
